# A monoclinic modification of propane-1,3-diyl bis­(pyridine-3-carboxyl­ate)

**DOI:** 10.1107/S1600536810054309

**Published:** 2011-01-08

**Authors:** Iván Brito, Javier Vallejos, Alejandro Cárdenas, Matías López-Rodríguez, Michael Bolte

**Affiliations:** a Departamento de Química, Facultad de Ciencias Básicas, Universidad de Antofagasta, Casilla 170, Antofagasta, Chile; b Departamento de Física, Facultad de Ciencias Básicas, Universidad de Antofagasta, Casilla 170, Antofagasta, Chile; c Instituto de Bio-Orgánica ’Antonio González’, Universidad de La Laguna, Astrofísico Francisco Sánchez N°2, La Laguna, Tenerife, Spain; d Institut für Anorganische Chemie der Goethe-Universität Frankfurt, Max-von-Laue-Strasse 7, D-60438 Frankfurt am Main, Germany

## Abstract

In the title compound, C_15_H_14_N_2_O_4_, (I), the mol­ecule lies on a twofold rotation axis which passes through the central C atom of the aliphatic chain, giving one half-mol­ecule per asymmetric unit. The structure is a monoclinic polymorph of the triclinic structure previously reported [Brito, Vallejos, Bolte & López-Rodríguez (2010). *Acta Cryst*. E**66**, o792], (II). The most obvious difference between them is the O/C/C/C—O/C/C/C torsion angle [58.2 (7)° in (I) and 173.4 (3)/70.2 (3)° in (II) for GG and TG conformations, respectively]. Another important difference is observed in the dihedral angle between the planes of the aromatic rings [86.49 (7)° for (I) and 76.4 (3)° for (II)]. The crystal structure features a weak π–π inter­action [centroid–centroid distance = 4.1397 (10)Å]; this latter kind of inter­action is not evident in the triclinic polymorph.

## Related literature

For conformation definitions, see: Carlucci *et al.* (2002 ▶). For the structure of the triclinic polymorph, see: Brito *et al.* (2010*a* ▶). For the synthesis and structural characterization of coordination polymers, see: Brito *et al.* (2010*b* ▶).
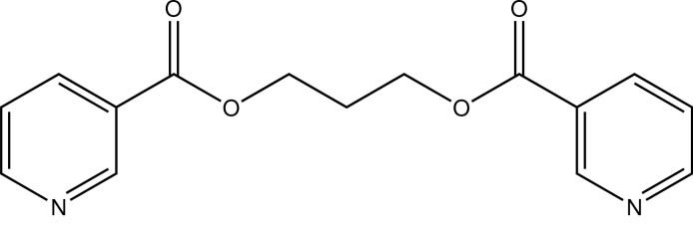


## Experimental

### 

#### Crystal data

C_15_H_14_N_2_O_4_
*M* *_r_* = 286.28
Monoclinic, 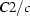

*a* = 24.414 (3) Å
*b* = 4.8328 (4) Å
*c* = 11.5667 (14) Å
β = 100.671 (10)°
*V* = 1341.1 (3) Å^3^
*Z* = 4
Mo *K*α radiation
μ = 0.11 mm^−1^
*T* = 173 K
0.35 × 0.33 × 0.13 mm

#### Data collection

Stoe IPDS II two-circle diffractometer
3193 measured reflections
1249 independent reflections
939 reflections with *I* > 2σ(*I*)
*R*_int_ = 0.037

#### Refinement

*R*[*F* ^2^ > 2σ(*F* ^2^)] = 0.033
*wR*(*F* ^2^) = 0.081
*S* = 0.92
1249 reflections
97 parameters
H-atom parameters constrained
Δρ_max_ = 0.17 e Å^−3^
Δρ_min_ = −0.14 e Å^−3^

### 

Data collection: *X-AREA* (Stoe & Cie, 2001 ▶); cell refinement: *X-AREA*; data reduction: *X-AREA*; program(s) used to solve structure: *SHELXS97* (Sheldrick, 2008 ▶); program(s) used to refine structure: *SHELXL97* (Sheldrick, 2008 ▶); molecular graphics: *XP* in *SHELXTL-Plus* (Sheldrick, 2008 ▶); software used to prepare material for publication: *SHELXL97*.

## Supplementary Material

Crystal structure: contains datablocks I, global. DOI: 10.1107/S1600536810054309/om2394sup1.cif

Structure factors: contains datablocks I. DOI: 10.1107/S1600536810054309/om2394Isup2.hkl

Additional supplementary materials:  crystallographic information; 3D view; checkCIF report

## Figures and Tables

**Fig. 1.**
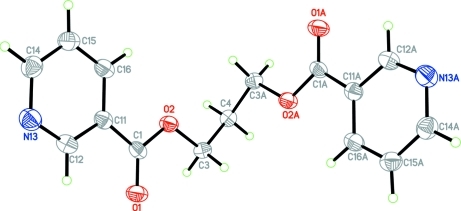
The structure of (I), showing the atom-numbering scheme. Displacement ellipsoids are plotted at the 50% probability level. [symmetry code: A = 1 - x,y,1/2 - z]

## supplementary crystallographic information

### Comment

This paper forms part of our continuing study of the synthesis and structural characterization of coordination polymers (Brito *et al.*, 2010*b*). We are particularly interested in the utility of the title compound of as a flexible ligand, and its binding modes, for the fabrication of different coordination polymers topologies. We report here the structure of a new polymorph of propane-1,3-diyl bis(pyridine-3-carboxylate) isolated during attempts to synthetize coordination polymers with silver trifluoromethanesulfonate of the ligand (Fig. 1, Table 1). In the title compound, (I) the molecule lies on a twofold rotation axis which passes through the central C atom of the aliphatic chain, giving one half-molecule per asymmetric unit. The structure is a monoclinic polymorph of the triclinic structure previously reported [Brito *et al.* (2010*a*). Acta Cryst. E66, o792], (II). There is excellent agreement between the geometric parameters of (I) and (II). The propanedyl group can adopt four possible conformations: *trans*-*trans* (TT), *trans*-*gauche* (TG), *gauche*-*gauche* (GG) and *gauche*-*gauche*' (GG') (Carlucci *et al.*, 2002).The most obvious difference between them is the O/C/C/C—O/C/C/C torsion angle [58.2 (7)° in (I) and 173.4 (3)/70.2 (3)° in (II) for GG and TG conformations, respectively]. Another difference between them is the angle between the planes of aromatic rings [86.49 (7)° for (I) and 76.4 (3)° for triclinic modification]. The crystal structure of the title compound has one intramolecular C—O··· H and one weak π–π interaction (4.1397 (10)Å *Cg*1— *Cg*1(i), symmetry code (i) =3/2 - *x*, 1/2 - *y* 1 - *z*; *Cg*1= N13/C12/C11/C16/C15/C14), whereas this last kind of interaction is not evident in the triclinic polymorph.The triclinic modification is less compact, as noted from the lower density (1.395 Mg m^-3^ compared with 1.418 Mg m^-3^ for the monoclinic form).

### Experimental

All reactions were carried out under an atmosphere of purified nitrogen. Solvents were dried and distilled prior to use. 5,5'-dinitro-2,2'-dithiodipyridine and silver trifluoromethanesulfonate were purchased from Aldrich. The title compound was obtained as colourless block crystals, in an attempt to prepare coordination polymers with silver trifluoromethanesulfonate and the ligand (II). The compound (I) was obtained by a mixture of (II) (1 mmol, 27.3 mg) and silver trifluoromethanesulfonate (1 mmol, 25.6 mg) in CH_3_CN (5 ml). The title compound was filtered off and washed with CH_3_CN. FT–IR (KBr pellet, cm^-1^): ν (w, C—H) 3086, ν(s, N=O of NO_2_ asymmetric) 1581, ν (v.s. of NO_2_ symmetric) 1352, ν(w, C—H disubstitution 1,4) 1962, ν(s, C—H disubstitution 1,4) 852, ν (w, C—N) 1101, ν(s, C=C) 1603, ν (w, C—H) 1010, (s, C=N) 1510, ν (w, C—S) 740, ν(w S—S) 552.

### Refinement

All H atoms could be located by difference Fourier synthesis but were ultimately placed in calculated positions using a riding model with C— H = 0.95 - 1.00 Å and with fixed individual displacement parameters [*U*_iso_(H) = 1.2 *U*_eq_(C)].

### Crystal data

**Table:**

C_15_H_14_N_2_O_4_	*F*(000) = 600
*M**_r_* = 286.28	*D*_x_ = 1.418 Mg m^−^^3^
Monoclinic, *C*2/*c*	Mo *K*α radiation, λ = 0.71073 Å
Hall symbol: -C 2yc	Cell parameters from 2928 reflections
*a* = 24.414 (3) Å	θ = 3.4–25.9°
*b* = 4.8328 (4) Å	µ = 0.11 mm^−^^1^
*c* = 11.5667 (14) Å	*T* = 173 K
β = 100.671 (10)°	Block, colourless
*V* = 1341.1 (3) Å^3^	0.35 × 0.33 × 0.13 mm
*Z* = 4	

### Data collection

**Table:**

Stoe IPDS II two-circle diffractometer	939 reflections with *I* > 2σ(*I*)
Radiation source: fine-focus sealed tube	*R*_int_ = 0.037
graphite	θ_max_ = 25.6°, θ_min_ = 3.4°
ω scans	*h* = −29→29
3193 measured reflections	*k* = −5→5
1249 independent reflections	*l* = −14→13

### Refinement

**Table:**

Refinement on *F*^2^	Secondary atom site location: difference Fourier map
Least-squares matrix: full	Hydrogen site location: inferred from neighbouring sites
*R*[*F*^2^ > 2σ(*F*^2^)] = 0.033	H-atom parameters constrained
*wR*(*F*^2^) = 0.081	*w* = 1/[σ^2^(*F*_o_^2^) + (0.0497*P*)^2^] where *P* = (*F*_o_^2^ + 2*F*_c_^2^)/3
*S* = 0.92	(Δ/σ)_max_ = 0.001
1249 reflections	Δρ_max_ = 0.17 e Å^−^^3^
97 parameters	Δρ_min_ = −0.14 e Å^−^^3^
0 restraints	Extinction correction: *SHELXL97* (Sheldrick, 2008), Fc^*^=kFc[1+0.001xFc^2^λ^3^/sin(2θ)]^-1/4^
Primary atom site location: structure-invariant direct methods	Extinction coefficient: 0.0099 (13)

### Special details

**Table:**

Geometry. All e.s.d.'s (except the e.s.d. in the dihedral angle between two l.s. planes) are estimated using the full covariance matrix. The cell e.s.d.'s are taken into account individually in the estimation of e.s.d.'s in distances, angles and torsion angles; correlations between e.s.d.'s in cell parameters are only used when they are defined by crystal symmetry. An approximate (isotropic) treatment of cell e.s.d.'s is used for estimating e.s.d.'s involving l.s. planes.
Refinement. Refinement of *F*^2^ against ALL reflections. The weighted *R*-factor *wR* and goodness of fit *S* are based on *F*^2^, conventional *R*-factors *R* are based on *F*, with *F* set to zero for negative *F*^2^. The threshold expression of *F*^2^ > σ(*F*^2^) is used only for calculating *R*-factors(gt) *etc*. and is not relevant to the choice of reflections for refinement. *R*-factors based on *F*^2^ are statistically about twice as large as those based on *F*, and *R*- factors based on ALL data will be even larger.

### Fractional atomic coordinates and isotropic or equivalent isotropic displacement parameters (Å^2^)

**Table:**

	*x*	*y*	*z*	*U*_iso_*/*U*_eq_	
O1	0.57094 (4)	0.5968 (2)	0.53846 (8)	0.0343 (3)	
C1	0.58554 (6)	0.5710 (3)	0.44442 (11)	0.0250 (3)	
O2	0.56326 (4)	0.7135 (2)	0.34794 (7)	0.0273 (3)	
C3	0.51676 (6)	0.8926 (3)	0.36128 (11)	0.0272 (3)	
H3A	0.5280	1.0184	0.4291	0.033*	
H3B	0.4849	0.7795	0.3759	0.033*	
C4	0.5000	1.0583 (4)	0.2500	0.0266 (5)	
H4A	0.4679	1.1804	0.2599	0.032*	
C11	0.62930 (6)	0.3744 (3)	0.42290 (11)	0.0254 (3)	
C12	0.65354 (6)	0.2013 (3)	0.51425 (12)	0.0299 (4)	
H12	0.6415	0.2180	0.5875	0.036*	
N13	0.69262 (5)	0.0130 (3)	0.50556 (10)	0.0342 (3)	
C14	0.70870 (6)	−0.0051 (3)	0.40112 (12)	0.0324 (4)	
H14	0.7368	−0.1360	0.3930	0.039*	
C15	0.68699 (6)	0.1548 (3)	0.30476 (12)	0.0317 (4)	
H15	0.6998	0.1327	0.2325	0.038*	
C16	0.64628 (6)	0.3481 (3)	0.31495 (11)	0.0290 (3)	
H16	0.6303	0.4601	0.2499	0.035*	

### Atomic displacement parameters (Å^2^)

**Table:**

	*U*^11^	*U*^22^	*U*^33^	*U*^12^	*U*^13^	*U*^23^
O1	0.0395 (6)	0.0414 (6)	0.0238 (5)	0.0043 (5)	0.0102 (4)	0.0026 (4)
C1	0.0281 (8)	0.0259 (7)	0.0199 (6)	−0.0060 (6)	0.0013 (6)	0.0007 (5)
O2	0.0313 (6)	0.0290 (5)	0.0220 (5)	0.0045 (4)	0.0062 (4)	0.0017 (4)
C3	0.0274 (8)	0.0293 (8)	0.0260 (7)	0.0015 (6)	0.0080 (6)	−0.0020 (6)
C4	0.0259 (11)	0.0260 (11)	0.0281 (9)	0.000	0.0057 (8)	0.000
C11	0.0268 (7)	0.0248 (7)	0.0238 (6)	−0.0053 (6)	0.0030 (6)	−0.0014 (5)
C12	0.0355 (9)	0.0308 (8)	0.0232 (6)	−0.0007 (7)	0.0049 (6)	−0.0009 (6)
N13	0.0378 (8)	0.0339 (7)	0.0303 (6)	0.0037 (6)	0.0045 (5)	0.0008 (5)
C14	0.0306 (9)	0.0315 (8)	0.0354 (8)	0.0008 (6)	0.0067 (6)	−0.0042 (6)
C15	0.0335 (8)	0.0346 (8)	0.0282 (7)	−0.0043 (7)	0.0091 (6)	−0.0037 (6)
C16	0.0321 (8)	0.0312 (8)	0.0231 (7)	−0.0052 (6)	0.0037 (6)	0.0009 (6)

### Geometric parameters (Å, °)

**Table:**

O1—C1	1.2122 (14)	C11—C16	1.3925 (16)
C1—O2	1.3381 (16)	C12—N13	1.3355 (19)
C1—C11	1.4849 (19)	C12—H12	0.9500
O2—C3	1.4585 (16)	N13—C14	1.3405 (17)
C3—C4	1.5069 (17)	C14—C15	1.379 (2)
C3—H3A	0.9900	C14—H14	0.9500
C3—H3B	0.9900	C15—C16	1.385 (2)
C4—C3^i^	1.5069 (17)	C15—H15	0.9500
C4—H4A	1.0042	C16—H16	0.9500
C11—C12	1.391 (2)		
			
O1—C1—O2	123.62 (13)	C16—C11—C1	123.47 (12)
O1—C1—C11	123.82 (12)	N13—C12—C11	124.20 (12)
O2—C1—C11	112.54 (10)	N13—C12—H12	117.9
C1—O2—C3	114.92 (9)	C11—C12—H12	117.9
O2—C3—C4	108.59 (9)	C12—N13—C14	116.40 (13)
O2—C3—H3A	110.0	N13—C14—C15	123.98 (14)
C4—C3—H3A	110.0	N13—C14—H14	118.0
O2—C3—H3B	110.0	C15—C14—H14	118.0
C4—C3—H3B	110.0	C14—C15—C16	118.94 (12)
H3A—C3—H3B	108.4	C14—C15—H15	120.5
C3^i^—C4—C3	115.77 (17)	C16—C15—H15	120.5
C3^i^—C4—H4A	108.4	C15—C16—C11	118.38 (13)
C3—C4—H4A	108.0	C15—C16—H16	120.8
C12—C11—C16	118.09 (13)	C11—C16—H16	120.8
C12—C11—C1	118.41 (11)		
			
O1—C1—O2—C3	−3.94 (19)	C16—C11—C12—N13	0.8 (2)
C11—C1—O2—C3	174.99 (11)	C1—C11—C12—N13	178.94 (14)
C1—O2—C3—C4	174.58 (12)	C11—C12—N13—C14	0.1 (2)
O2—C3—C4—C3^i^	58.11 (8)	C12—N13—C14—C15	−0.7 (2)
O1—C1—C11—C12	2.7 (2)	N13—C14—C15—C16	0.4 (2)
O2—C1—C11—C12	−176.23 (13)	C14—C15—C16—C11	0.5 (2)
O1—C1—C11—C16	−179.25 (13)	C12—C11—C16—C15	−1.1 (2)
O2—C1—C11—C16	1.83 (19)	C1—C11—C16—C15	−179.13 (13)

Symmetry codes: (i) −*x*+1, *y*, −*z*+1/2.

### Hydrogen-bond geometry (Å, °)

**Table:**

*D*—H···*A*	*D*—H	H···*A*	*D*···*A*	*D*—H···*A*
C12—H12···O1	0.95	2.51	2.8298 (18)	100

## References

[bb1] Brito, I., Vallejos, J., Bolte, M. & López-Rodríguez, M. (2010*a*). *Acta Cryst.* E**66**, o792. 10.1107/S1600536810008810 PMC2983924 21580631

[bb2] Brito, I., Vallejos, J., Mundaca, A., Cárdenas, A., Albanez, J., Vargas, D. & López-Rodríguez, M. (2010*b*). *Mol. Cryst. Liq. Cryst.* **521**, 158–167.

[bb3] Carlucci, L., Ciani, G., Proserpio, D. M. & Rizzato, S. (2002). *CrystEngComm*, **22**, 121–129.

[bb4] Sheldrick, G. M. (2008). *Acta Cryst.* A**64**, 112–122. 10.1107/S0108767307043930 18156677

[bb5] Stoe & Cie (2001). *X-AREA* Stoe & Cie, Darmstadt, Germany.

